# Present Application and Perspectives of Organoid Imaging Technology

**DOI:** 10.3390/bioengineering9030121

**Published:** 2022-03-16

**Authors:** Keyi Fei, Jinze Zhang, Jin Yuan, Peng Xiao

**Affiliations:** State Key Laboratory of Ophthalmology, Zhongshan Ophthalmic Center, Guangdong Provincial Key Laboratory of Ophthalmology and Visual Science, Sun Yat-Sen University, Guangzhou 510060, China; feiky@mail2.sysu.edu.cn (K.F.); zhangjz5@mail2.sysu.edu.cn (J.Z.)

**Keywords:** organoid, imaging technology, microscopy, optical coherence tomography

## Abstract

An organoid is a miniaturized and simplified in vitro model with a similar structure and function to a real organ. In recent years, the use of organoids has increased explosively in the field of growth and development, disease simulation, drug screening, cell therapy, etc. In order to obtain necessary information, such as morphological structure, cell function and dynamic signals, it is necessary and important to directly monitor the culture process of organoids. Among different detection technologies, imaging technology is a simple and convenient choice and can realize direct observation and quantitative research. In this review, the principle, advantages and disadvantages of imaging technologies that have been applied in organoids research are introduced. We also offer an overview of prospective technologies for organoid imaging. This review aims to help biologists find appropriate imaging techniques for different areas of organoid research, and also contribute to the development of organoid imaging systems.

## 1. Introduction

An organoid is a type of three-dimensional cell culture. Organoids can be generated either from pluripotent stem cells or from adult stem/progenitor cells, which can differentiate into multiple organ specific cell types. More importantly, organoids have a spatial structure similar to the specific organs and can reproduce some of their functions; thus, the in vivo tissue’s physiology can be reproduced in vitro [1,2,3,4]. In short, organoids are in vitro simplified model systems of organs. Compared with the traditional 2D culture system, the advantage of organoids is that they better resemble the natural organs in gene expression, microscale tissue architecture and metabolic function [5]. In recent years, scientists have successfully established organoid models of the intestine [6,7,8], stomach [9,10], prostate [11,12], kidney [13,14,15], brain [16,17,18], retina [19,20,21], pancreas [22,23,24], breast [25,26,27], liver [28,29,30], thyroid [31,32], salivary gland [33,34], endometrium [35,36], taste buds [37,38] and airways [39,40,41]—either from healthy or cancerous tissues. Organoids have become an important tool for fundamental biological research on embryonic development, tissue structure, biological function, cell metabolism, disease simulation, drug selection and regenerative medicine.

The development of organoids is closely related to the development of imaging technology. Using an ordinary optical microscope to image the biological sections that are immunohistochemical or that have been immunolabeled, we can roughly observe the 2D tissue structure and the distribution of single or multiple markers. However, 2D imaging cannot meet the needs of organoids research, because it cannot interpret the complex 3D structure of organoid. Fortunately, with the 3D imaging technologies and novel volume imaging methods that have developed rapidly in recent years, the 3D structure of whole-mount tissues can be characterized across the scales from cell to tissue [42,43]. Compared to traditional sample-sectioning 2D imaging, 3D imaging has advantages in visualizing the complexity of biological specimens. Three-dimensional imaging with superior qualities is essential for understanding the cellular composition, cell shape, cell–cell interactions and cell fate of intact biological samples [44]. With the in-depth study of organoids, 4D living imaging, which studies dynamic processes at high resolution, plays a significant role in real-time observation, tissue dynamics and physiological progress [45,46,47].

As organoid culture methods developed and an increasing number of organoid applications, organoid imaging has become a fundamental part of physiological and pathological studies. This review aims to provide an overview of the current imaging technologies and subsequently identify the prospects in imaging for organoids.

## 2. Imaging Technology

The imaging process of cultured organoids can be simply summarized as the restoration of organoids from their 3D matrix, fixation and immunolabeling, optical clearing and then 3D rendering with visualization software after imaging [44]. The two main analyses of these organoid images are quantification (counting cells and their components, measuring the labeling intensity or advanced quantification of specific areas) and morphological measurement (size, shape, etc.) [48]. The following introduces the commonly used imaging technologies and expounds their advantages, disadvantages and applications. Table 1 summarizes these technologies.

### 2.1. Electron Microscopy (EM)

**Figure 1:**
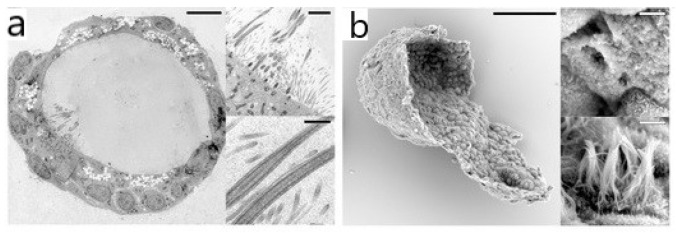
(**a**) TEM image of airway organoid cross section showing the epithelium structure, and details display apical microvilli and cilia with their characteristic microtubule structure. (**b**) SEM image of an airway organoid visualizes its 3D architecture, as well as basal and apical ultrastructure. Details display apical surfaces of secretory and multi-ciliated cells. (**a**,**b**) are reproduced from [41].

EM can achieve nanometer resolution [49]. Transmission electron microscopy (TEM) and scanning electron microscopy (SEM) are two commonly used types of EMs that have been widely applied for observation of the organoid ultrastructure. TEM has excellent performance in imaging the details of organoids’ structure stratification, for example, showing the epithelial structure of human airway organoids [41] (see Figure 1), the typical hepatocyte structure of liver organoids [30], the microstructure of NPC EVs (extracted vectors derived from neural progenitor cells) [50], etc. Due to the ultra-fine resolution of TEM, characteristic structures were found which could not be realized by optical microscopy. In a human cerebral organoid model of CLN3-JNCL (juvenile neuronal ceroid lipofuscinosis), the disease characteristics, i.e., the increased presence of autophagic vacuoles, can be observed by TEM [51]. TEM imaging showed the emergence of a ribbon synapse between photoreceptors and bipolar cells in the inner plexiform layer, which suggests the functional maturation of retinal organoids [21,52,53]. However, the sample preparation of TEM imaging is extremely complex, and ultra-thin sections (usually 50~100 nm) must be prepared. SEM does not need ultra-thin samples, the depth of field is dozens of times larger than TEM and the image can reveal the three-dimensional topography and distribution of features by observing the sample from various angles [54]. SEM interpreted the 3D imaging of the epithelial structure in human airway organoids [41] and kidney tubular epithelial organoids [55]. The limitation of SEM is that only the surface structure of the sample can be observed.

The use of EM has the disadvantages of causing great damage to the sample, only observing the sample surface, and being unable to realize the real-time and dynamic observation of living cells. It is not suitable for the observation of biological samples, especially living samples.

### 2.2. Bright-Field Microscopy

Bright-field microscopy can be used to observe the shape of organoids [56] and measure two-dimensional parameters, such as length and area [57]. Samples do not require any particular preparation. Organoid cultures can be examined without staining and the illumination will not alter the true colors of the samples. Bright-field microscopy images are also used to measure the overall size with automatic methods [58,59,60]. Indeed, the size is evaluated by diameter [56,61,62], perimeter [63] or surface area [51,64], which can evaluate organoids’ growth or can be used to compare different culture groups (such as healthy and disease models). In bright-field microscopy, the perimeter is measured on the boundaries of manual or semi-automated selected regions and the diameter is measured the longest distance between two points of a selected region. These measures need to be operated in the early stage of development due to the shape heterogeneity in the later stage in their culturing model [48]. Bright-field microscopy can also carry out the real-time and dynamic detection of living cells. The culture and differentiation process (at least 175 days in vitro) of retinal organoids is observed by using bright-field microscopy, and three different morphological stages of the development process of hPSC-derived retinal organoids can be identified through this process [21]. This system is simple and practical for panoramic observation, but it is also accompanied by the sacrifice of fine and comprehensive structural details because of its no more than 2 µm in-plane resolution and two-dimensional nature; it can only realize partial shape measurement because it cannot capture three-dimensional information. Another problem is that the light source of bright-field microscopy emits below the sample, and the contrast is generated by the light absorption in the sample. When the contrast is too high, the observation quality will decrease, resulting in image distortion. On the contrary, when the contrast is too low, most cells are invisible due to non-staining.

### 2.3. Fluorescence Microscopy

Fluorescence microscopy [65] uses a certain wavelength of light as the light source to irradiate the sample to make it emit fluorescence, and then observes the shape and position of the object under the microscope. Fluorescence microscopy is widely used in the structural and functional imaging of biological samples. Some substances in cells, such as NADPH [66], can emit fluorescence after ultraviolet irradiation; in addition, some substances themselves cannot emit fluorescence, but if they are stained with fluorescent dyes or fluorescent antibodies, they can also emit fluorescence by ultraviolet irradiation, so that specific substances can be imaged and located. Fluorescence microscopy is the tool for qualitative and quantitative research on these substances with a spatial resolution of tens to hundreds of nanometers and a temporal resolution in milliseconds to seconds.

**Figure 2:**
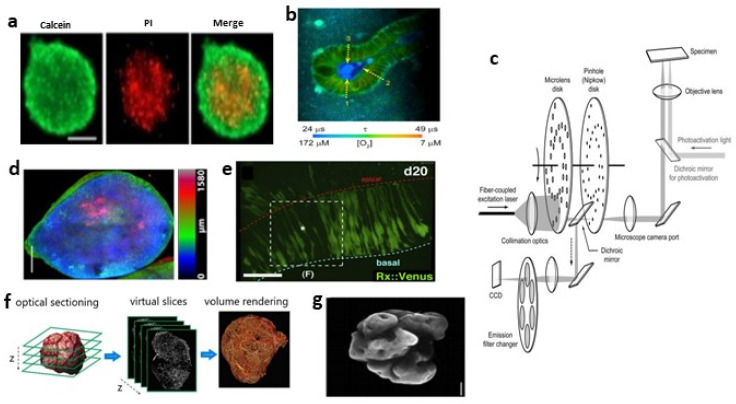
(**a**) WFFM was used to observe viable and dead cells in the cancer spheroid. Stained with calcein-AM (green) and propidium iodide (PI, red). Reproduced from Reference [81] under the Creative Commons License (CC BY 4.0); (**b**) FLIM imaging revealed presence of O2 micro-gradients between basal and apical membranes in resting organoids. Reprinted from Biomaterials, 146, D.B.; Okkelman, I.A.; Foley, T.; Papkovsky Dmitriev R.I., Live cell imaging of mouse intestinal organoids reveals heterogeneity in their oxygenation, 86–96,Copyright (2022), with permission from Elsevier; (**c**) Schematic drawing of SDC microscopy. Reprinted from Methods in Enzymology, 504, Stehbens, S.; Pemble, H.; Murrow, L.; Wittmann, T., Imaging intracellular protein dynamics by spinning disk confocal microscopy, 293–313, Copyright (2022), with permission from Elsevier; (**d**) Color-coded SDC image of endogenous GFP in a human cerebral organoid. Adapted from [84]. (**e**) Multiphoton images showing interkinetic nuclear migration of retinal progenitors in the day-20 hESC-derived optic vesicle epithelium. Reprinted from Cell Stem Cell, 10, Nakano, T.; Ando, S.; Takata, N.; Kawada, M.; Muguruma, K.; Sekiguchi, K.; Saito, K.; Yonemura, S.; Eiraku, M.; Sasai, Y., Self-formation of optic cups and storable stratified neural retina from human ESCs, 771–785, Copyright (2022), with permission from Elsevier; (**f**) Schematic drawing of light sheet 3D reconstruction. Reprinted from Neoplasia, 16, Dobosz, M.; Ntziachristos, V.; Scheuer, W.; Strobel, S. Multispectral fluorescence ultramicroscopy: Three-dimensional visualization and automatic quantification of tumor morphology, drug penetration, and antiangiogenic treatment response, 1–13, Copyright (2022), with permission from Elsevier; (**g**) Light sheet image of a 6-week-old human cerebral organoid. Reprinted from Cell Stem Cell, 20, Li, Y.; Muffat, J.; Omer, A.; Bosch, I.; Lancaster, M.A.; Sur, M.; Gehrke, L.; Knoblich, J.A.; Jaenisch, R. Induction of Expansion and Folding in Human Cerebral Organoids, 385–396, Copyright (2022), with permission from Elsevier.

**Figure 3:**
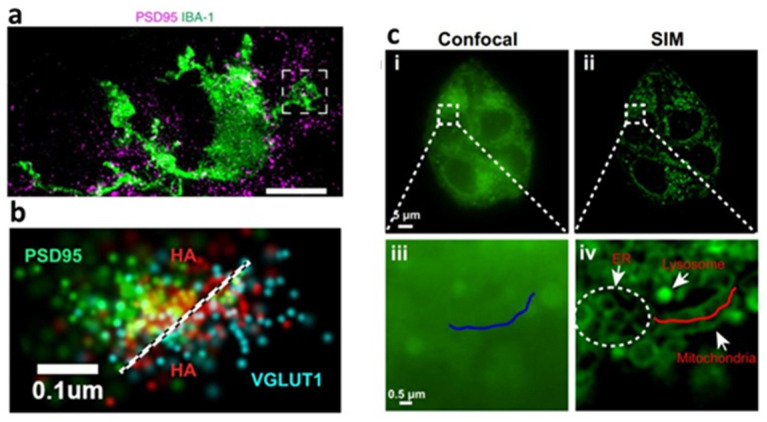
(**a**) STED microscopy showed the distribution of microglia (IBA-1) in relation to the postsynaptic marker PSD95 in cerebral organoids. Reproduced from Reference [92] under the Creative Commons License (CC BY 4.0); (**b**) STORM was used to visualize hyaluronan (HA) at individual excitatory synapses in 3D cortical spheroid (presynaptic marker vGlut-1, blue; HA, red; postsynaptic marker PSD95, green). Reproduced from Reference [140] under the Creative Commons License (CC BY 4.0); (**c**) Confocal (**i**) and (**iii**) and SIM (**ii**) and (**iv**) images for the NapBu-BPEA-stained (Zn2+ fluorescent probe) HeLa cells. Reproduced from Reference [141] under the Creative Commons License (CC BY 4.0).

The main advantage of fluorescence imaging is that with the introduction of probes and markers, it can visualize tissue, cell type, particular biomolecules and gene expression with great specificity. However, invasive fluorescence is to be used in most of the studies, resulting in no further utilization of samples after imaging. Other limitations in fluorescence live-cell imaging are photobleaching and phototoxicity, which are mainly induced by excited fluorophores [67,68,69]. Excited fluorophores can generate reactive oxygen species (ROS) that react with easily oxidizable components, leading to the loss of the fluorescence signal (photobleaching) [70] and cell cycle arrest or cell death (phototoxicity) [71,72]. The effective strategy to reduce photobleaching and phototoxicity is to minimize the excitation light dose [73,74]. However, with the decrease in the excitation light dose, the image quality will deteriorate (decreased signal-to-noise ratio (SNR)) [75]. Consequently, balancing the excitation light dose has become a major challenge in live-cell imaging. Figure 2 and Figure 3 show the imaging of organoids by different fluorescence microscopes.

#### 2.3.1. Wide-Field Fluorescence Microscopy (WFFM)

The resolution of WFFM [76] is higher than bright-field microscopy, which is more suitable for the study of cell biology. In this instrument, parallel beams illuminate the entire sample (or wide field of view) simultaneously to excite (via the filter block) the fluorophore it contains. WFFM is widely used in organoid imaging with immunofluorescence and immunohistochemical labeling [25,27,39,40,63,77,78,79] because it is advantageous for quickly imaging an entire organoid and images can be easily captured with a camera. Cell viability can be evaluated by measuring diameter, perimeter and fluorescence intensity, thus making it useful for screening drug response [80,81,82] (see Figure 2a). However, the fluorescence signal is not fully quantitative, and despite attempts to optimize staining and analysis methods [83], it is still not suitable for the objective quantification of the drug response.

In WFFM imaging, although the microscope only images the focal plane of the objective, the whole sample will be illuminated by the excitation light, which not only greatly increases the photobleaching, but more seriously, the WFFM cannot restrict these signals to enter the detector, making it impossible to achieve depth sectioning when imaging thick samples, thus limiting the visualization of three-dimensional organoids. In addition, images with low contrast and spatial resolution may be produced due to diffraction-limited optics and inevitable out-of-focus light. By reducing the objective numerical aperture (NA) [85], the loss of image contrast due to out-of-focus light can be compensated to some degree, but the image resolution will also be reduced. The lateral resolution of wide-field microscopy is about 200–300 nm, and the axial resolution is about 500–700 nm [86]. The axial resolution can be increased to 200–300 nm by structural illumination and subsequent image processing [87,88].

#### 2.3.2. Laser Scanning Confocal Microscopy (LSCM)

LSCM is the most widely used instrument for organoids imaging [48], which produces optical sections by scanning the sample point-by-point with a laser beam focused on the sample and uses pinholes to exclude defocused background fluorescence from detection [85]. Thus, it allows for image depth-specific slices of organoids with a penetration depth ≈100 µm and 3D imaging.

Organoid images obtained by LSCM are analyzed to measure various parameters at the subcellular level, such as intensity [41,89], shape [30,90], surface [91], cell distribution [17] or for three-dimensional reconstruction [26,55,56]. By counting cells, biomarkers and nuclei, we can quantify the activity of organoids and track their growth and development [18,92,93,94,95]. LSCM can also be used in many disease studies, for example, observing the invasion of cerebral organoid glioblastoma [96], comparing the number and function of synapses in cerebral organoid models of different diseases [51,97], determining disease markers [98], setting fluorescence intensity thresholds to quantify tumor infiltration areas [99], etc. Four-dimensional real-time living cell imaging has also been widely used, for example, for measuring the area [57] or volume [100] of specific areas to monitor the morphological changes of organoids, observing the construction of retinal organoids in real time [101], observing the chromosomal segregation defects of tumor organoids [45,46], studying the cytodynamics of organoids [102], etc. In addition, combined with fluorescence lifetime imaging microscopy (FLIM), it can be used to analyze the metabolism of organoids. For example, Okkelman I.A. et al. [103] revealed the oxygenation of individual mouse intestinal organoids and analyzed their metabolic heterogeneity (see Figure 2b).

LSCM uses a very small laser beam (point light source) to scan the image point-by-point and line-by-line, illuminating only a small point of the sample at any time, resulting in a long acquisition time. Although confocal microscopy can block defocus signals with a pinhole, these signals still exist and produce photobleaching. In addition, due to the small diameter of the pinhole and the short exposure time of each part of the sample, the laser source illumination intensity needs to be increased to compensate for the reduced image resolution, which further increases photobleaching and phototoxicity. The light scattering of the sample has a great influence on LSCM, resulting in a small penetration depth. The issue of fluorescence crosstalk occurs when multiple indicators are used. For these reasons, some teams prefer to use light-sheet microscopy for 3D culture imaging, although it requires a longer and more complex sample preparation protocol [48]. In recent years, the resolution of LSCM has been further improved, enabling high-resolution imaging (lateral resolution of 120 nm and axial resolution of 350 nm, compared to 250 and 800 nm for conventional confocal microscopy). This opens up the possibility of studying intracellular processes in organoids, such as vesicle transport or cytoskeleton remodeling [44].

#### 2.3.3. Spinning Disc Confocal (SDC) Microscopy

Traditional LSCM uses point-by-point scanning and a photomultiplier tube (PMT) as a detector, resulting in a slow acquisition speed and the requirement of increased illumination intensity, which leads to great photobleaching and phototoxicity and is not suitable for living cell imaging. However, SDC microscopy [104,105,106] can realize the rapid imaging of living cells. The principle of SDC microscopy is to place a Nipkow disk on the object’s image plane, and the disk is distributed with helically arranged pinholes (see Figure 2c). The laser light source covers the range of all pinholes (i.e., the scanning area). When the disk spins, the pinholes are scanned in turn, so as to realize the complete scanning of the sample. SDC microscopy can achieve theoretical frame rates of up to 2000 frames s^−1^. Compared with the traditional point scanning method, the multi-point synchronous scanning method not only greatly improves the acquisition speed, but also means that an area array camera (back illuminated metal oxide conductor (CMOS) cameras or electron multiplying charge-coupled devices (EMCCD)) can be used to replace PMT and improve quantum efficiency, so as to reduce the excitation power and greatly reduce the photobleaching and phototoxicity to the samples.

Based on the advantage of rapidly acquiring images of SDC microscopy, it is widely used in the high-throughput imaging of organoids, so as to reach statistical significance. Lukonin, I. et al. [107] developed an image-based organoid screening platform using approximately 10^7^ images collected by SDC microscopy to describe the phenotypic landscape of organoid development and infer functional genetic interactions. When SDC microscopy is used for 3D remodeling [19,20,84,91,93,108], the images have higher quality, high-resolution observation and localization of living cells, more accurate cell counting and morphometric parameters, such as cell volume and sphericity (see Figure 2d). However, it cannot scan arbitrary 3D shapes across the sample. The 4D real-time imaging of SDC microscopy can observe cell division, cytoskeleton rearrangement, cell migration, etc. [109,110]. The main disadvantage of SDC microscopy is the pinhole crosstalk effect [111]; that is, some defocused emitted light will be transmitted to the camera through the adjacent pinholes, which will produce a perceptible fuzzy background signal and affect the axial resolution of the image. Therefore, compared with the single-point laser scanning system, its theoretical confocal degree is reduced. In addition, the pinhole size cannot be adjusted to change the optical slice strength and imaging resolution.

#### 2.3.4. Multiphoton Microscopy

LSCM excites fluorophores with ultraviolet or a visible single photon; however, multiphoton microscopy (including two-photon) [112,113] uses pulsed infrared laser sources, which emit more than one low-energy photon at a time to excite fluorophores. Although the energy of any of these low-energy photons is insufficient to excite an electron, their combined energy is enough to lift an electron to the excited state, and thus, stimulate fluorescence. Infrared light has stronger penetration (penetration depth of up to 1 mm) than visible light in biological tissue, so multiphoton microscopy can better solve the problem of the tomography of deep substances in biological tissue and expand the scope of application. Multiphoton excitation is a non-linear process with accurate localization characteristics; that is, only photons at the focal point can excite fluorophores. Photobleaching and phototoxicity are limited to the vicinity of the focal point, which is conducive to reduce the autofluorescence and light scattering of samples for further improving the image clarity, so that living cells can be observed for a longer time. Different from LSCM, multiphoton microscopy does not rely on pinholes, so the measurement of the light path is simpler, more reliable and effective.

By the nuclear segmentation of the same organoid without light clearing, Dekkers et al. [44] found that LSCM and multiphoton microscopy had similar nuclear resolution on the surface of an organoid, but multiphoton microscopy performed better at deeper layers. Multiphoton microscopy also has excellent performance in organoids using 4D real-time imaging [19,90,108] (see Figure 2e). Optical metabolic imaging (OMI) is a type of multiphoton microscopy to probe the intrinsic fluorescence intensities and lifetimes of metabolic enzymes, which can assess changes in the cellular metabolism of organoids and be applied to drug screening research [114,115,116]. Multiphoton microscopy can also combine hyperspectral imaging (HSpec) and FLIM to judge cell activity and specific molecular localization through fluorescence intensity [107], so as to evaluate organoid development [117]. Intravital multiphoton microscopy (IVPM) [118,119] enables the study of dynamic processes at the single-cell level, allowing the real-time and spatial observation of pathology, but only superficial tissues can be imaged. The image acquisition of multiphoton microscopy is often painstakingly slow for point-by-point scanning along the entire sample. A new method, single-objective planar-illumination two-photon microscopy, was designed [120], combining a two-photon system with a spinning disk, which permits one order of magnitude faster imaging and a wide-field.

#### 2.3.5. Light Sheet Fluorescence Microscopy (LSFM)

The difference between the LSFM [121,122,123] and the traditional microscope lies in the illumination mode of excitation light. By creating a thin excitation light sheet that is orthogonally oriented to the detection axis, only the samples of the focal plane are illuminated, whereas the samples above and below are unaffected (see Figure 2f). The fluorescence signal is collected by a detection objective mounted perpendicular to the illumination axis, which are decoupled from illumination. By moving or rotating the sample, large samples can be imaged and photographed from multiple angles to obtain a complete 3D image. Equipped with high quantum efficiency detectors, such as EMCCD or CMOS cameras, LSFM greatly improves the imaging speed and image SNR. LSFM has experienced the development from SPIM (selective/single plane illumination microscopy) to DSLM (digital scanned light-sheet microscopy) to LLSM (lattice light-sheet microscopy). Especially the LLSM, which replaced the conventional Gaussian beam with the Bessel beam [124], enables volumetric imaging of samples at high spatiotemporal resolution and 3D in vivo imaging with minuscule levels of photobleaching and phototoxicity [47]. Recently, the combination of adaptive optics with LLSM (AO-LLSM) [125,126] corrects tissue-induced aberrations caused by the inhomogeneous refractive index of multicellular specimens, which can further improve spatial resolution and signal-to-background ratios.

The application of LSFM in organoid imaging proves that it has become a broad-spectrum and powerful tool in cell and developmental biology. The surface area and volume of the 3D reconstruction of light-sheet microscopic organoid images can be performed [127,128] (see Figure 2g), making it possible to measure the thickness of specific structures [129]. LSFM can realize the automatic quantification of multiple tumor parameters and the three-dimensional visualization of drug penetration at the cellular level [130]. Favreau et al. [114] demonstrated that SPIM is a powerful tool for the high-throughput screening of organoid treatment response by determining endogenous fluorescence from NAD(P)H and FAD. Hundreds of pancreas and cholangiocarcinoma organoids were imaged in parallel using LSFM for up to 7 days and identified and quantified the organoid morphogenesis process at cellular resolution [131]. However, LSFM requires samples to be completely cleared, and obstacles such as bubbles may cause shadows in the path of the illumination [132]. In addition, compared with LSCM and multiphoton microscopy, the subcellular resolution obtained by LSFM is still suboptimal. Furthermore, LSFM generates huge terabytes of data during recording and measurement, so it is necessary to equip a device that can provide appropriate storage and data analysis support.

#### 2.3.6. Super-Resolution Fluorescence Microscopy

The spatial resolution of conventional fluorescence microscopy is limited by Abbe’s law [133], and the best resolution is limited to about 200–300 nm in the lateral dimensions and 500–800 nm in the axial dimensions. However, super-resolution fluorescence microscopy can overcome the diffraction barrier and obtain nanoscale resolution in living cell imaging [134]. With the improvement of spatial resolution by an order of magnitude, when it is used in organoid imaging, it can observe the previously unsolved details of cell structure, and even study biological processes at the single-molecule level. Here, we will mainly discuss three super-resolution microscopies: the first is STED microscopy based on stimulated emission quenching, the second is PALM/STORM based on single-molecule localization and the third is SIM based on structured light illumination imaging.

(1) Stimulated Emission Depletion (STED) Microscopy

The STED [135] super-resolution imaging technology is based on LSCM. The difference is that STED microscopy adds the depletion light on the basis of the excitation light, which overlaps the two laser sources and allows the depletion of fluorescence, so that the effective excitation spot size is greatly reduced. The resolution of STED microscopy is about 50 nm in the lateral dimensions (can be improved to below 40 nm) and 100 nm in the axial dimensions. The most basic application of STED microscopy is to image the intensity distribution of fluorescent samples, which successfully realize the intensity imaging and quantification of fluorescent nanoparticles and fluorescent-labeled organoid samples, such as imaging calcium’s signal intensity in cerebral organoids [136], the maximum intensity projection of specific marker proteins in the microglial of cerebral organoids [92] (see Figure 3a), the quantification of relative fluorescence intensity per cell in intestinal organoids [137], etc. In addition, it can quantify the subcellular structural details, such as nuclear volume measurement in proliferating tumor spheroids [138], the thickness measurement of the apical F-actin belt [63] and thin-layer neuroepithelial structure [139] in cerebral organoids, etc. STED microscopy can also be used for FLIM and the fluorescence correlation spectrum (FCS) measurement of fluorescent samples, which makes its application in organoid imaging more extensive.

Compared with other types of super-resolution microscopy, STED microscopy has relatively fast imaging speed, no need for specialized fluorophores, no need to computationally reconstruct images and has more accurate molecular localization because of photons provision by laser. However, the main limitations include the fact that a high laser intensity will lead to serious photobleaching and phototoxicity, vibration must be considered, and that there is a slight improvement in *z*-axis resolution compared to LSCM [142]. Fortunately, STED can be combined with other imaging techniques to overcome some limitations. The combination of two-photon excitation (TPE) and STED microscopy can extend the depth penetration [143]. TPE-STED is especially suitable for imaging within scattering tissue media. The combination of SPIM and STED microscopy can improve the axial resolution [144]. Due to the significant crosstalk between different fluorophores, it is difficult to realize multicolor STED microscopy, but the selection of special dyes can solve this problem [145].

(2) Single-Molecule Localization Microscopy (SMLM)

Photoactivated localization microscopy (PALM) [146] and stochastic optical reconstruction microscopy (STORM) [147] are two single-molecule localization super-resolution imaging techniques with the help of special fluorophores. PALM utilizes photoactivatable fluorophores, whereas STORM utilizes photoswitchable dyes, both of which have two states of “activation” and “deactivation”. When different fluorescent probes are activated at different time points, the fluorophores can be imaged without spatial overlap and can be located with high precision. The position of fluorescent probes can be determined by iterated activation, and then the super-resolution image can be reconstructed according to the position of a large number of probes [134]. PALM and STORM have been used in the imaging of the internal workings and structures of the cellular environment, as well as the extracellular-related components, such as determining the nanoscale clustering of cell membrane receptors (Wnt receptor, LRP6, and EGFR) in colonic organoids [148], detecting hyaluronan between the pre- and post-synaptic compartment of synapse in 3D cortical spheroids [140] (see Figure 3b), etc.

PALM and STORM can provide excellent spatial resolution (∼20 nm lateral resolution and 50 nm axial resolution) [146,147], so they can be used for single-molecule imaging, which is very important for quantitative imaging. However, one of the limitations is the low temporal resolution, which means image acquisition takes a long time. In addition, they need special fluorophores, and the quality of fluorophores and sample preparation are highly essential to generate excellent images, the same as the extensive processing after image acquisition. PALM and STORM have less localization ability than STED. When the sample is irradiated by a short-wavelength and an iterative excitation light, it brings serious photobleaching and phototoxicity, and generates a large number of free radicals to damage the living cell. Therefore, it is difficult to apply PALM and STORM to live-cell imaging. Moreover, due to the strong scattering and absorption of thick tissues, STORM and PALM are limited to thin samples (<a few µm) [149].Considering the above reasons, PALM and STORM are not widely used in organoid imaging. In order to achieve 3D imaging of SMLM, Bon P et al. [150] utilized self-interference to realize the 3D super-resolution imaging of organoids’ endogenous proteins and the 3D tracking of fluorescent-labeled submicron structures (e.g., molecules, vesicles or other organelles), but this technique has the limitation of resolution uncertainty.

(3) Structured Illumination Microscopy (SIM)

SIM [151] utilizes multiple interference beams with a specially structured pattern to illuminate fluorescent-labeled samples. Both the fluorescence generated by fluorophores and the overlying illumination produce moiré patterns. Moving and rotating patterned illumination can capture multiple images and can then decode the high-resolution information in these images by deconvolution to reconstruct fine details. Compared with traditional fluorescence microscopy, the resolution of SIM has about a two-fold improvement, with a resolution of 100–150 nm in the XY direction. In addition, 3D SIM is realized by using three beams of interference light which generate a three-dimensional modulation pattern, with a resolution of ~300 nm in the Z direction [88]. Furthermore, saturated structure illumination microscopy (SSIM) is a variant of SIM, which has up to a five-fold improvement in resolution by using non-linear patterned excitation [152]. However, serious photobleaching and phototoxicity limit its application in living cell imaging.

Although the resolution of SIM is lower than that of STED and SMLM, SIM requires less illumination intensity than SMLM and has a lower requirement for wavelength than that of STED, which makes it particularly suitable for the living cell super-resolution imaging of organoids. Moreover, the imaging acquisition time of SIM is short, so it is suitable for 3D imaging. Applications of SIM in organoid cell biology include structural imaging and dynamic observation. Super-resolution imaging of lysosomes in liver organoids uses special lysosomal fluorescent probes and real-time tracking of lysosomal motion in living cells [153], and the super-resolution localization of cellular endogenous proteins in intestinal organoids [154]. Fang H et al. [141] used SIM and Zn^2+^ fluorescent probes, allowing super-resolution morphology-correlated organelle identification in living cells and tracking events in specific organelles within organoids (see Figure 3c). SIM improves the resolution by optical means, so it is compatible with the fluorophore and labeling protocol developed by traditional fluorescence microscopy. Subdiffraction multicolor imaging with 3D SIM can be used to locate different molecules or structures in the 3D cellular environment [155]. However, the drawback of SIM is that the image processing is time-consuming and may produce artifacts [142]. Moreover, in thick and densely labeled samples, severe scattering can dramatically reduce image quality. SIM captures dozens of images to build a single super-resolution frame, and the requirement for multiple frames increases the risk of cumulative phototoxicity [156]. The combination of 3D-SIM and SPIM has been proven to limit fluorescence near the focal plane, which can greatly reduce photobleaching and phototoxicity, improve time resolution and increase penetration depth [124,157].

In recent years, other super-resolution imaging technologies have begun to emerge in organoid imaging. Cone diffraction microscopy (CODIM) has been used to observe the localization and transport of opsins in retinal organoids [158]. CODIM [159,160] is a structural illumination technology that uses conical diffraction to generate illumination patterns. The main advantage of this technique is that it can realize long term low phototoxicity and the photobleaching of super-resolution imaging and the tracking of living cells, which benefits from the need for only one scanning system and a low light dose on the sample plane. In addition, the newly designed NIR Bessel-beam emission saturation nanoscopy (NIRB) [161] can be used for the super-resolution mapping of single nanoparticles in 3D multicellular spheroids. By utilizing a “non-diffractive” doughnut-shaped Bessel beam and near-infrared excitation, NIRB realizes low phototoxicity imaging in highly scattering tissues. This method can maintain sub-100 nm resolution located at a depth of over 50 µm inside spheroids, with a smaller excitation power and faster imaging speed.

### 2.4. Optical Coherence Tomography (OCT)

**Figure 4:**
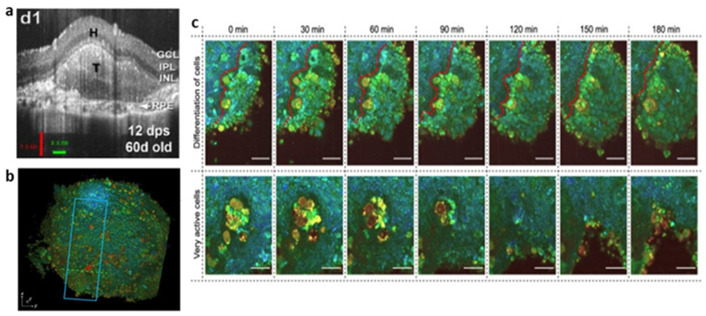
(**a**) In vivo development of retinal organoid transplant monitored by OCT. Reproduced from Reference [171] under the Creative Commons License (CC BY 4.0); (**b**,**c**) D-FFOCT 3D image (**b**) differentiation process is shown in the top row and the cell’s dynamic active region is shown in the bottom row (**c**) of hiPSC-derived retinal organoids. Reproduced from Reference [173] under the Creative Commons License (CC BY 4.0).

OCT [162] is an imaging technology based on laser interferometry. By detecting the amplitude and echo time delay of backscattered near-infrared light from samples, the cross-sectional imaging of the microstructure inside the tissues is carried out. OCT can provide micron-scale resolution (about 10 µm) and a high penetration depth range of about 1–3 mm for tissue imaging [163]. At the same time, the non-invasive nature of OCT enables it to conduct longitudinal research in the same sample, without the requirement for the resection and processing of tissue samples. Based on the advantages of living imaging, long-term tracking, label-free and 3D imaging, OCT has become a hot topic in organoid imaging in recent years. First, OCT provides excellent organoid visualization and can remodel over time. Chhetri, R.K. et al. [164] used spectral-domain OCT (SD-OCT), equipped with an ultra-high resolution and short acquisition-time, to image normal breast organoids and tumor breast organoids to observe and quantify their morphological differences. Compared with wide-field microscopy and fluorescence microscopy, OCT enhances the ability to volumetrically image individual organoids [165], provides unbiased insight into the internal structure of organoids [166] and further reveals the unique anatomical characteristics of organoids not observed by other technologies [21]. In addition, OCT has been used in organoids to monitor cell movement and extracellular matrix invasion, enabling the visualization and quantification of tissue dynamics, such as subcellular motility through the statistical analysis of rapid-time-sequence signals at the same location [167]. For example, the in-place motions (motility) of mammary epithelial cells within organoids was evaluated and quantified [168], to analyze the viability of tumor organoids [169], etc. With the continuous improvement and optimization of this function, the OCT method is often utilized in the study of drugs or treatment schemes. Moreover, without destructive processing or exogenous labels, OCT has proven to be a valuable tool for monitoring transplantation growth. When retinal organoids are transplanted into the subretinal or vitreous space of animal models, the quality of retinal organoids can be predicted by quantitative and volumetric analysis using SD-OCT [170,171,172] (see Figure 4a).

The limitations of OCT include that it is not suitable to observe the internal structure of transparent samples, the distal shadow when laser penetrates the samples and the image variability in the scanning laser direction [174,175]. There is a contradiction between the imaging depth and resolution of OCT; for example, enhancing the lateral resolution requires increasing the numerical aperture (NA), which will reduce the depth of field, resulting in only a small layer of clear imaging. Therefore, the lateral resolution of the classical OCT system is about 10 µm, which is not enough to analyze the cell structure. In order to meet the requirement of en face images, full-field OCT (FFOCT) was proposed.

FFOCT is able to achieve better than 1-µm axial (Z) resolution and 0.5-µm transverse (XY) resolution by utilizing high NA objectives in a Linnik interferometer configuration [176]. Different from traditional OCT, which requires transverse scanning of the illumination spots on the tissue’s surface, FFOCT uses the full-field illumination of a spatial incoherent light source, high-speed megapixel camera as detector array and time-domain phase modulation to obtain an en face view at a designated depth [177,178]. Although FFOCT provides superior resolution to image the complex structure of living tissue, it lacks functional information. Dynamic full-field OCT (D-FFOCT) [179] is developed on the basis of FFOCT by measuring temporal fluctuations of the backscattered light. The colored images generated based on dynamic measurement can generate endogenous contrast related to organelle motility [180], and have submicron spatial resolution and millisecond temporal resolution. D-FFOCT offers astounding opportunities to track the evolution of the same organism at different developmental stages, without the requirement of using exogenous markers or destructive methods and allows imaging of different layers at multiple depths while maintaining the integrity of the sample. Using this analysis, Leroux C.E. et al. [181] successfully separated the fractions of slow and fast dynamics in tumor spheroids. A series of retinal organoids imaged by D-FFOCT at consecutive steps of development showed the cellular process, such as progenitor cell proliferation and migration, cell type differentiation including the evolution of the boundary between neural and non-neural retinal cells and the evolution of the organoid into layered retina [173] (see Figure 4b,c). Although the penetration depth of FFOCT is less than OCT, and the penetration depth of D-FFOCT is typically ten times less than FFOCT, D-FFOCT can still reach the penetration depth of 100 µm [182], which is equivalent to LSCM. Unfortunately, there are few examples of using FFOCT and D-FFOCT imaging in organoid research. We look forward to the further demonstration of the application of this technology within the visualization of non-invasive tissue dynamics in the future.

### 2.5. Others

By imaging retinal organoids with phase contrast microscopy, limited microstructure details can be displayed [117], and retinal organoid transplantations can be selected according to transparency and morphological criteria [170]. The advantage of phase contrast microscopy [183] is that it can continuously observe living cells without staining. The basic structure of phase contrast microscopy is the same as that of ordinary optical microscopy with a low resolution, and the halo will hinder the observation of delicate structures. Moreover, phase contrast microscopy cannot be used to observe thick samples, which should be prepared with a thickness of 5 µm or thinner.

Micro-computed tomography (microCT) can be used to demonstrate the mineralization of dentin-pulp-like organoids and realize 3D morphometric analysis through algorithm reconstruction [184]. When applied to retinal organoids, microCT can distinguish between the retinal neuroepithelium and hollow acellular area in the center of organoids, allowing the quantitatively evaluation of the thickness of the neuroepithelium [117]. MicroCT can provide high-resolution (5 µm) 3D information for complex structures, but it is limited to fixed tissues and cannot image living cells.

Total internal reflection fluorescence microscopy (TIRFM) [185] is not widely used in organoid studies, because the dynamic range it can observe is usually less than 100 nm, which limits its application to only the cell surface. However, TIRFM has a spatial resolution below 100 nm and provides high-quality images and reliable observation data of micro-structures and single molecules. Moreover, TIRFM illuminates only a very thin layer, which reduces its photobleaching and phototoxicity compared with the traditional fluorescence microscopy. TIRFM has excellent advantages in single-protein dynamics research, such as the real-time single-molecule imaging of NGS Wnts molecules in organoid growth models [186] and the live-cell imaging of epithelial cells in intestinal organoid models with the tracking of enzyme substrate molecules, membrane proteins and actin [154]. Additionally, a microtubule gliding assay was performed in a retinal organoid disease model in which the microtubule velocity was quantified [187].

## 3. Discussion

Organoids, models with similar structures and functions to real organs, have provided a wide range of utilities in biological research in recent years. It is worth noting that although organoids achieve a high level of tissue heterogeneity, not all cell types can be reproduced in organoids, and the extent of their maturation in vitro is unclear [188]. Therefore, it is necessary to monitor the differentiation process of organoids in real-time. In order to achieve this, most experiments have to carry out gene analysis, biochemical analysis, Western blot analysis, immunofluorescence imaging and other molecular biology technologies for organoids, and analyze them at the gene, protein and functional levels. Zhou et al. [189] used flow cytometry, gene expression analysis and immunostaining to analyze the differentiation of different cell types and the expression of related proteases in airway organoids. However, these techniques are destructive to the tested samples, which cannot continue to be used in following experiments, let alone in practical applications. In addition, due to the heterogeneity of pluripotent stem cells, even organoids from the same batch cannot be guarantee to have the same characteristics as the sacrificial samples [190]. We urgently request a relatively non-invasive method to monitor and evaluate organoids; imaging technology may become a breakthrough in this area.

The complex three-dimensional organ-like structure of organoids puts forward high requirements for imaging technology. On the one hand, it is necessary to treat organoids as whole objects for 3D imaging and minimize photobleaching and phototoxicity. On the other hand, the structure and function of organoids need to be displayed at the cellular level so as to evaluate their integrity as a biological model. The ideal imaging technology is combined with a high frame rate, a large field of view and low photobleaching and phototoxicity to achieve three-dimensional high spatial resolution and cell-level functional analysis, as well as the ability to meet the requirements of real-time and non-invasive imaging. Fortunately, scientists are working hard in this direction. Zong et al. [191,192] designed a miniaturized two-photon microscopy with an improved speed (40 Hz) and spatial resolution (0.64 µm) and utilized it to resolve activity at single dendritic spines in freely moving animals. Huang et al. [193] developed a new Hessian SIM for the rapid, long-term, super-resolved imaging of moving vesicles or loops in the endoplasmic reticulum with a spatiotemporal resolution of 88 nm and 188 Hz. Loyez, J. et al. [194] developed a self-interference (SELFI) microscopy for three-dimensional, real-time super-resolution imaging and single-particle tracking, which has been proved from cells to living organ sections.

However, each imaging technology has its own limitations. Traditional wide-field fluorescence microscopy has a low resolution and no optical sectioning capability, so it is not suitable for the three-dimensional imaging of organoids. LSCM and two-photon microscopy can be used to image the three-dimensional structure of organoids, but their low image acquisition rate, high photobleaching and phototoxicity limit their long-term imaging of organoids. SDC microscopy and LSFM improve the image acquisition rate, reduce photobleaching and phototoxicity and allow imaging at a deeper sample depth. However, their resolution is inferior to that of LSCM, and their practical application is limited by the more complex sample preparation and higher costs involved. Although super-resolution fluorescence microscopy overcomes diffraction obstacles and obtains a spatial resolution of a few tens of nanometers, it offers a small field of view, a very low imaging depth and a slow imaging rate. Interestingly, the combined use of multiple imaging techniques can compensate for their respective defects and maximize efficiency. The combination of two-photon excitation mode and light-sheet microscopy is the most classic, which has proved that the imaging depth is twice as deep than single-photon light-sheet microscopy, and the imaging speed is more than ten times faster than point-scanning two-photon microscopy with negligible photobleaching [195,196,197]. Shin, Y. et al. [198] designed an oblique scanning two-photon LSFM (OS-2P-LSFM). The system overcomes the limitation of highly constrained specimen geometry that LSFM requires by using a remote focusing technique and improves the scattering limitation of the light sheet by using a two-photon Bessel beam. Therefore, OS-2P-LSFM allows the robust 3D imaging of organoids at high speed, without translation and scattering. Nguyen T.D. et al. [199] developed a single-objective multiphoton light-sheet microscopy (SO-MP-LSM), which achieves a 270 nm lateral resolution of and 800 nm axial resolution in organoids, and provide a high imaging speed and a deeper imaging depth. The SO-MP-LSM is compatible with multi-well plates, which allow high-throughput drug screening using organoid or spheroid models. Browne, A.W. et al. [117] used a variety of imaging techniques including phase contrast microscopy, OCT, FLIM and HSpec, to image retinal organoids. Multimodal real-time imaging shows the dynamic changes in the structure, differentiation and metabolism in retinal organoids without damaging the growth of organoids. The significant advantage of the combined multiple technologies is the non-destructive observation of organoid morphology and function based on naturally occurring fluorophores.

At present, most organoid imaging needs immunofluorescence labeling, which may bring about the following problems [44]: (1) the fluorescent antibody signal is weak, so it is necessary to amplify the signal with secondary antibody steps; (2) due to the inefficient penetration to the nucleus, it shows the weak signal of immunolabeled nucleoprotein; and (3) the fixation time also affects the fluorescent signal, so it is necessary to optimize the fixation time in the process of sample preparation. We definitely cannot deny the continuous development of fluorescent labeling technology, especially the development of gene fusion fluorescent protein tags [200,201,202,203], which makes it possible to use smaller, brighter and more permeable fluorescent probes for single-molecule imaging. However, the processed organoids will no longer be utilized for therapeutic purposes, which limits the exploration of organoid-related substitutive therapy. It has become a trend to develop imaging technologies that do not have the requirement for fluorescent labeling and that can be tracked for a long time.

Combining the principles of light-scattering spectroscopy (LSS) with LSCM, confocal light absorption and scattering spectroscopic (CLASS) microscopy was fabricated [204]. CLASS microscopy possesses a spatial resolution comparable to super-resolution fluorescence microscopy but without destruction. There is no requirement of exogenous labels, which can avoid their potential interference with cell processes. Therefore, it is suitable for living cells and tissues and enables observation of the function of cells and organelles at scales in the order of 100 nm. Subsequently, a coherent version of the CLASS microscopy was developed [205], which can correct the limitation in which CLASS microscopy lost its capability for high-NA optics. With an NA = 1.3 objective lens, the spatial resolution of the coherent CLASS microscopy can be improved to 10 nm, and it can be used for almost any light scattering spectroscopic application using lens. Thanks to the nanoscale resolution of the CLASS system, it was used to detect the dynamic distribution of chromatin during organoid differentiation without sacrificing or even modifying the samples [190]. However, the 3D image construction ability of CLASS microscopy is not excellent and the field of view can only reach 200 µm, which limits its application in relatively large organoids.

FFOCT and D-FFOCT, as mentioned above, are the other two excellent potential imaging techniques for the label-less longitudinal research of organoids. There is no requirement for a fixing or cleaning procedure which may disturb 3D structures, such as by causing the shrinking and collapsing of organoids [44]. The dynamic signal of FFOCT is generated by the intrinsic motion within cells with a 1 µm axial resolution, 0.5 µm transverse resolution, up to 1 mm depth and a 1260µm × 1260 µm field of view [182]. D-FFOCT not only inherits the advantages of FFOCT but also reveals the information of cell function at the subcellular level. Based on these advantages, we can speculate about the feasible application scenarios of FFOCT and D-FFOCT: (1) To establish a high-throughput real-time imaging platform to quickly test the drug response and toxicity of organoids. The high-throughput platform enables us to study the changes in cell morphology and activity in organoids with a high resolution during drug screening in vitro. (2) Continuously detect organoids-on-a-chip and multi-organoid microfluidic systems, then establish a biological library with the organoid’s microfluidic system, which will display the unique “fingerprint” of various organoids. (3) The long-term monitoring and evaluation of the growth and differentiation process in organoids that are anticipated to be applied in transplantation or product substitution therapy. Real-time imaging can enhance the effective utilization of organoids as drug screening tools, development and disease models and cell substitution therapy sources. At present, image-based cell analysis techniques are being developed to identify, segment and quantify densely arranged cells in large tissue volumes [206,207] or quantify phenotypic differences among various cell populations with high-throughput image analysis tools [208,209]. These techniques can be combined with FFOCT to improve research efficiency. Many researchers are working to identify cells via D-FFOCT contrast through machine learning algorithms, with the ultimate goal of completely removing the need for labeling [173].

In conclusion, the development of imaging technology affects the progress of organoid research to a great extent. Different imaging technologies have their own advantages and disadvantages. Scientists can choose appropriate imaging technologies or combine different imaging technologies according to the purpose of the research topic. We expect that the innovation of imaging technology in the future will promote the application of organoids in various studies and even replace animal models.

## Figures and Tables

**Table 1:** The commonly used imaging technologies for organoids.

Technology	Resolution	Penetration Depth	3D	Living Cell Imaging	Photobleaching/Phototoxicity	Advantage	Disadvantage	Application
EM	~0.1 nm	~150 nm	±	−	−	Nanometer resolution	Damage sample	Precise observation of ultrastructure
Bright-field microscopy	~2 µm	−	−	+	−	No staining is required Organoid differentiation can be dynamically observed	3D information is not captured	Measurement of 2D parameters Monitoring of organoid culture process
WFFM	200–300 nm (XY) 500–700 nm (Z)	Bad	−	+	Low	Rapid imaging Low cost Simple operation	Low resolution No 3D imaging	Measurement of 2D parameters Cell viability is judged by fluorescence intensity
LSCM	≈200 nm (XY) 350–800 nm (Z)	≈100 µm	+	+	High	High resolution 4D imaging	Long acquisition time Small penetration depth Increased illumination intensity	Measure various parameters at the subcellular level Analyze metabolism of organoids combined with FLIM
SDC microscopy	<LSCM	>LSCM	+	+	Lower than LSCM	Rapid imaging (2000 frames s-1) 4D imaging	Pinhole crosstalk effect Cannot scan arbitrary 3D shapes	High-throughput imaging of organoids 3D remodeling
Multiphoton microscopy	≈LSCM	Hundreds of µm	+	+	Low, restricted to focal plane	Stronger penetration Pinhole independent Accurate location	Long acquisition time High cost	Long time tracking observation Evaluation of cellular metabolic changes
LSFM	<LSCM	>Multiphoton microscopy	+	+	Low, restricted to focal plane	Rapid imaging High signal-to-noise ratio	Complex sample preparation Thin light sheet limits small field of view	Suitable for large sample imaging Long-term imaging of living samples 3D reconstruction
STED	50 nm (XY) 100 nm (Z)	<50 μm	+	Not suitable	High	No need for specialized fluorophores Less postprocessing needed Relatively fast imaging than other super-resolution fluorescence microscopies	High laser intensity leads to serious photobleaching and phototoxicity	Quantification of structural details Intensity imaging and quantification of fluorescent molecule Observation of molecular movements
SMLM	20 nm (XY) 50 nm (Z)	<a few of μm	−	−	Serious than STED	Ultra-high resolution Precise location of fluorophores	Long acquisition time Requirement of special fluorophores Extensive postprocessing needed	Cellular submicron structure imaging Quantification of single fluorophores
SIM	100–150 nm (XY) ~300 nm (Z)	<50 μm	+	+	Low	Conventional fluorophores can be used Short acquisition time Lower cost than other super-resolution microscopes	Image processing is time consuming and may produce artifacts Severe scattering in thick samples	Tracking the movement of subcellular structures in living cells Simultaneous localization of different molecules or structures
OCT	10 µm	1–3 mm	+	+	−	Non-invasive No need to process samples Long-term tracking	Low resolution Contradiction between the imaging depth and resolution Not suitable to transparent samples	Volumetrically image individual organoid Visualization and quantification of tissue dynamics Monitoring transplantation growth
FFOCT	0.5 µm (XY) 1 µm (Z)	1 mm	+	+	−	Non-invasive High resolution Short acquisition time	Lack of functional information	Imaging of cell structure Tracking different developmental stages of samples
D-FFOCT	0.5 µm (XY) 1 µm (Z)	100 µm	+	+	−	Non-invasive Obtain cell function information	Penetration depth is less than FFOCT Cannot acquire large volume quickly	Cell dynamics analysis Tracking different developmental stages of samples
